# User perceptions about sharing exposure notification information for communicable diseases

**DOI:** 10.3389/fdgth.2022.926683

**Published:** 2022-07-28

**Authors:** Benjamin Schooley, Sue S. Feldman

**Affiliations:** ^1^College of Engineering and Computing, Univeristy of South Carolina, Columbia, SC, United States; ^2^Department of Health Services Administration, University of Alabama at Birmingham, Birmingham, AL, United States

**Keywords:** mobile platforms, privacy, pandemic response, exposure notification, contact tracing, communicable disease response

## Abstract

**Background:**

The (GuideSafe™) Exposure Notification System (ENS) was built and deployed in (Alabama) for anonymous sending and receiving of COVID-19 exposure alerts to people who have been in close contact with someone who later reports a positive COVID-19 test. Little is known about how the demographic groups perceive recent privacy-preserving the ENS innovations, including their usability, usefulness, satisfaction, and continued interest in sharing COVID-19 exposure information. The purpose of this study was to investigate how users across the demographic groups perceive the sharing of exposure information with various types of organizations and to investigate how end-user perceptions of the ENS usability, usefulness, and satisfaction differ across the demographic groups within the context of a statewide deployment of an exposure notification system.

**Methods:**

A survey was administered to (state residents blinded for review) (*N* = 1,049) to assess propensity to share COVID-19 infection data and evaluate end-user perceptions about usability, usefulness, and satisfaction with the (Alabama) ENS. The ANOVA and the Tukey's Honestly Significant Difference (HSD) *post-hoc* tests were conducted to assess the demographic group differences.

**Results:**

The ENS survey participants had a high awareness of contact tracing, exposure notifications, and the (GuideSafe™) ENS and reported having downloaded the app. Survey results revealed the majority of participants rated the app as useful (*n* = 490, 79%), easy to use (*n* = 490, 79%), and reported satisfaction with its use (*n* = 546, 88%). Other results suggest that ethnicity and age may be important factors for trust in sharing exposure information.

**Conclusion:**

The (GuideSafe™) system was one integrated component of comprehensive education and work re-entry strategy across (Alabama) that reached a broad user base. Users across the different demographic groups perceive the sharing of information about their communicable disease exposures differently. Furthermore, demographic factors play a role in which types of organizations individuals are willing to share their communicable disease exposure information. Public health institutions, employers, schools, healthcare providers, and technology designers may want to consider these findings as they construct technologies and perform outreach campaigns aimed at reducing infection rates with the ENS and related technologies.

## Background and significance

Contact tracing (CT) is a public health response launched during an outbreak that identifies infected individuals and seeks out others they have been in close physical contact to mitigate the spread of infection (1). Central to the purpose of CT and exposure notification is the need for disease containment, accomplished *via* identification of infected individuals, isolation for ~10–14 days, and continuous monitoring of potential symptom development (2). Manual CT relies on the memory and honesty of each infected person (3). Much like that which is seen in sexually transmitted diseases, people were not always forthcoming with close contact during COVID-19 CT (4, 5).

During the COVID-19 pandemic, disease spread overwhelmed public health departments that traditionally conduct CT and many recognized that technology could potentially provide viable solutions to fill this gap (6). Technology enabled the Exposure Notification System (ENS), which is a system by which each person carries an app on their phone. This app senses and logs close contact with another phone also carrying the app. Should one of these people later test positive for COVID-19 and use the app to report the test, all the close contacts are anonymously notified. Population uptake of CT apps is essential for effective CT, though the degree of population penetration required has been debated (7). While there are many different systems in the United States, the (Alabama) (GuideSafe™) ENS utilized the Google Apple Exposure Notification (GAEN) application programming interface (API). The interoperability functionality on both the Android and iOS platforms and the privacy-preserving nature factored heavily into our app selection. The GAEN was released as an API in May 2020 to state jurisdictions, primarily health departments. The premise of the GAEN is a privacy preserving, completely anonymous system to provide exposure notifications sooner than manual case CT. Through low-energy Bluetooth, phones in close contact exchange encrypted data. The data live on the phone and do not get transmitted to a central location, thereby preserving the privacy of each user. Each state customized the GAEN and many included situational guidance, such as quarantine information, symptom assessment tool, and testing locations.

The ENS literature has reported mixed results on its benefits. While stronger primary research on the effectiveness of ENS is needed, most studies support that a combination of manual CT and the ENS is effective—with the ENS providing important benefits. The ENS benefits include a reduction in secondary cases, improved case detection over manual efforts during an epidemic (8, 9) and the ability to save personnel time, improve accuracy with large data sets, and be easier to transport compared with paper forms when implemented in low- to middle-income countries (8). Simulation studies have shown significant benefits, with one finding the potential to prevent up to 80% of all the transmissions (10). Less privacy-preserving techniques in Korea (11) and Singapore (12) have reported success to aid in lowering infections, as has a privacy-preserving method employed in the Netherlands (13).

Low usage of the ENS due to lack of usability, usefulness, or trust likely results in low participation, leading to low notification rates and creating an information gap for users expecting to receive notifications. Several factors have been associated with the lack of mass adoption of the ENS apps for pandemics, including an individuals' awareness and acceptance (12), technology usability (13), level of innovativeness and social influence (14), level of interest in constant or continuous use of the same app, level of user trust (15), perceptions about health benefits (16), and very different user expectations across a large, diverse, and heterogeneous population (17). Furthermore, users may have varying motivations for using an ENS app, including intrinsic motivation to help others (15, 16), compliance with rules/regulations for fear of not being able to work or participate in desirable activities (12, 14), fear of illness or death (15, 16), and others. By and large, public resistance to the ENS has focused on user trust and app usefulness contrasted by the potential infringement of privacy (16, 18–25). Privacy and security risks may lead to low app performance, user fears about using the service, or distrust in results (21). These risks are real, with Dar et al. (26) detailing a variety of potential attacks that could occur for the Bluetooth- or geolocation-based ENS solutions such as resource drain, trolling, replay attacks, or ransomware (23). Technical threats and challenges affect human perceptions about the usability, utility, and security of the ENS solutions. Although previous research has documented factors associated with the mass adoption of the ENS apps during pandemics (26), to date, there is limited literature specifically examining how the various demographic groups may adopt these innovative ENS solutions to enhance traditional CT methods over the course of statewide deployment during the COVID-19 pandemic.

### Regional context

A Kaiser Family Foundation analysis placed (Alabama) at just over 43% of adults 18 years and over for being at a higher risk of serious illness from COVID-19, placing (Alabama) first of the deep south states at risk of developing a serious COVID-19-related illness (27). Considering the volume, velocity, and reach of COVID-19, lead health informatics researchers at (name of University blinded for review purposes) surmised that traditional contact tracing methods were not going to be sufficient for the (Alabama) Department of Public Health (XDPH) or for the privacy concerned population in (Alabama). Thus, the team explored privacy-preserving technology-enabled CT that was soon renamed the Exposure Notification Systems (ENSs) (Alabama) is in the deep south with a 2019 population of almost 5 million people. Demographically, whites comprise 69% and blacks almost comprise 27%, with all the other races under 5% each^1^. Statistics on smartphone users in the state that can handle the (GuideSafe™) app suggest that there are just under 1.9 million households in (Alabama) with just over 89% of those having a smartphone^2^. The system went live on 3 July 2020 and continues to operate at the time of manuscript submission. As of 1 October 2021, 310,000 (GuideSafe™) downloads had occurred and 1,512 verified positive tests had been reported. Outside the scope of this study, but noteworthy, is that the XDPH estimates that (GuideSafe™) led to earlier exposure notifications by approximately 4 days and that traditional contact tracing only identified 1–2 close contacts when the overall average is thought to be around 8 people.

### Objectives

With various factors affecting how innovation is designed and implemented, there may be some features that are helpful for some demographic groups and in conflict with the needs of other groups making the one-size-fits-all approach challenging (17). Therefore, there are two primary objectives of this study: to investigate how users across the different demographic groups perceive the sharing of COVID-19-related exposure information with various types of organizations involved in the ENS implementation and to investigate how end-user perceptions of three mHealth app constructs (the ENS usability, usefulness, and satisfaction) differ across the demographic groups within the context of a statewide deployment of the one Exposure Notification System across (Alabama).

## Methods

### Data

The initial study sample was obtained from users who participated in helpbeatcovid19.org, a regional COVID-19 initiative, and who agreed to participate in COVID-19 research.

Text message survey invitations were sent to mobile phone numbers from users of helpbeatcovid19.org who previously agreed to participate in the reserach. Two text reminders were sent 10 days apart. Users were instructed to download and use the (GuideSafe™) privacy preserving the ENS implemented in (Alabama).

### Survey administration

Using Qualtrics online survey platform, a survey was administered to potential (GuideSafe™) users across the state of (Alabama) from 3 June to 30 June 2021. While the survey responses were anonymous, each respondent could provide contact information for a chance to receive a $100 Amazon gift card survey incentive. Twenty gift cards were randomly distributed among the respondents providing contact information. Of the 12,934 messages that were sent, 443 (3.4%) messages did not go through resulting in 12,491 text messages sent. A total of 1,049 (8%) participants responded to the survey. Participants that did not complete the survey were excluded from the final study sample.

### Measures

The survey administered was a combination of survey items that were adapted from and used with permission from Lincoln Laboratory, Massachusetts Institute of Technology (communication dated 26 January 2021) and survey items from the Mhealth App Usability Questionnaire (MAUQ) (28) to assess survey participant experiences using the (app)™ ENS app (see Supplemental Material for survey instrument). Survey questions were asked using a Likert scale from 1 to 5, with 1 being the most positive and 5 being the most negative and 3 being neutral. The Likert scale was changed from the MAUQs original 7-point scale to a 5-point scale to be consistent with the rest of the survey instrument and to facilitate ease of use for respondents. Simulation studies and empirical studies have generally concurred that reliability and validity are best with either 5- or 7-point scale and that between three different scale formats (5, 7, and 10 points), there appear to be no appreciable differences in terms of standard variation, skewness, or kurtosis, suggesting that all are equally valuable for ANOVA/regression analysis (29, 30). Cronbach's alpha was used to assess the internal consistency of the MAUQ survey and its three construct subscales were documented at 0.895 for ease of use and satisfaction, 0.829 for system information arrangement, and 0.900 for usefulness, indicating strong internal consistency (28). In addition, strong construct validity and criterion validity were documented for the MAUQ survey (28).

### Data analysis

The ANOVA was conducted to assess differences between gender, race/ethnicity, education, and age groups. The ANOVA default model (SSI) controls for the imbalance of sample size. The Tukey's Honestly Significant Difference (HSD) *post-hoc* tests were conducted to assess significant differences between the groups where other than dichotomous responses were collected. The analyses were conducted using IBM SPSS. Results show significance at the *p* < 0.05 level.

## Results

Gender, age, education level, and race/ethnicity demographics are shown in Table 1, representing the total number of completed demographic questions. A total of 838 participants answered the ENS information sharing questions, while a total of 621 participants reported using the (GuideSafe™) app and answered the MAUQ usability questions.

**Table 1:** Study sample demographic results.

**Study Sample** **Demographics**		***N***	**%**
Gender	Female	523	62.4%
	Male	294	35.1%
	Other	21	2.5%
Age	18–36	212	25.4%
	37–49	207	24.8%
	50–61	223	26.7%
	>61	194	23.2%
Education	High School diploma or GED	36	4.3%
	Some College	161	19.3%
	Completed College	320	38.4%
	Master's Degree	174	20.9%
	Terminal Degree	143	17.1%
Race/Ethnicity	White	675	80.5%
	African American	80	9.5%
	Other	52	6.2%
	Asian	31	3.7%

The following sections discuss the ANOVA and Tukey's results, which are shown in Table 2.

**Table 2:** The ANOVA results by question by demographic.

	**Age**		**Education**		**Gender**		**Ethnicity**	
	**F**	***P***	**F**	***P***	**F**	***P***	**F**	***P***
**Please indicate how likely you are to download GuideSafe if one of the below people or organizations asks you to.**								
Healthcare provider	0.312	0.817	0.103	0.981	1.047	0.351	3.648	**0.012** ^**^
Your employer or school	1.981	0.115	1.063	0.374	5.232	**0.006** ^**^	2.139	**0.094** ^*^
City, county, or state public health dept	1.734	0.159	1.652	0.159	0.927	0.396	1.641	0.179
Federal public health authorities (eg., CDC)	1.038	0.375	1.420	0.226	1.216	0.297	1.436	0.231
App store provider (eg., Google, Apple)	2.692	**0.045** ^**^	6.706	**0.001** ^**^	0.743	0.476	3.756	**0.011** ^**^
**If you were to test positive for COVID-19 today, how likely are you to let GuideSafe notify other service users if one of the below people or organizations asks you to?**								
Healthcare provider	0.892	0.445	0.114	0.978	0.786	0.456	2.444	**0.063** ^*^
Your employer or school	2.604	**0.051** ^**^	0.379	0.824	4.570	**0.011** ^**^	1.959	0.119
City, county, or state public health dept	0.130	0.942	0.485	0.747	1.624	0.198	2.140	**0.094** ^*^
Federal public health authorities (such as the CDC)	0.021	0.996	0.647	0.629	2.904	**0.055** ^*^	2.09	0.100
App store provider (eg., Google, Apple)	6.591	**0.001** ^**^	4.26	**0.002** ^**^	1.474	0.23	1.776	0.150
**Please indicate how likely you are to let GuideSafe send your exposure notifications to the following individuals or organizations.**								
Healthcare provider	2.262	**0.080** ^*^	0.789	0.533	0.223	0.800	1.062	0.365
Your employer or school	4.738	**0.003** ^**^	0.492	0.741	2.618	0.074	3.510	**0.015** ^**^
City, county, or state public health dept	0.13	0.942	0.221	0.927	1.854	0.157	0.975	0.404
Federal public health authorities (eg., CDC)	0.834	0.475	0.200	0.938	1.343	0.262	0.929	0.426
App store provider (eg., Google, Apple)	4.991	**0.002** ^**^	5.209	**0.001** ^**^	0.234	0.791	1.275	0.282
	F	P	F	P	F	P	F	P
Ease of Use	2.117	**0.097** ^*^	2.823	**0.024** ^**^	0.347	0.707	6.339	**0.001** ^**^
Interface and Satisfaction	0.078	0.972	2.415	**0.048** ^**^	2.017	0.134	2.023	0.131
Usefulness	0.666	0.573	4.091	**0.003** ^**^	1.451	0.235	3.764	**0.011** ^**^

*F = F value.*

*P = P value.*

** *p < 0.05.*

* *p < 0.1*.

### Usability perceptions

In terms of gender and age, there were no statistically significant differences between groups on satisfaction, usefulness, and ease of use at the *p* < 0.05 level. There was an effect of education level on ease of use [*F*_(4, 613)_ = 2.823, *p* = 0.024]; however, Tukey's results showed only a mild difference at the *p* < 0.1 level.

There was also an effect of ethnicity on ease of use [*F*_(3, 618)_ = 6.339, *p* ≤ 0.001], with Tukey revealing statistically significant differences between white (M = 1.836, SD = 0.887, *p* = 0.008), African American (M = 1.470, SD = 0.530), and other ethnicities (M = 1.409, SD = 0.622) at the *p* < 0.05 significance level. African Americans had a lower mean score than others meaning that they rated the app easier to use than the other groups.

There was a statistically significant difference of education level on app satisfaction [*F*_(4, 613)_ = 2.823, *p* = 0.048], with the Tukey's *post-hoc* test showing that those with a high school diploma or general education diploma (GED) (M = 1.323, SD = 0.651, *p* = 0.079) rated the app mildly lower (higher satisfaction) than those reporting having a Master's degree education (M = 1.729, SD = 0.921) at *p* < 0.1.

There was a statistically significant effect of race on app usefulness [*F*_(3, 616)_ = 3.764, *p* = 0.011], with the Tukey's *post-hoc* analysis revealing differences between white (M = 1.876, SD = 0.893, *p* = 0.035) and African Americans (M = 1.557, SD = 0.702); African Americans rating the app as more useful than whites.

Education had a significant impact on app usefulness [*F*_(4, 611)_ = 4.091, *p* = 0.003]. The Tukey's *post-hoc* test indicated a difference between those with a Master's degree (M = 2.047, SD = 0.969, *p* = 0.014) and those with a high school diploma (M = 1.479, SD = 0.715), Master's degree (M = 2.047, SD = 0.969, *p* = 0.029) and those with some college experience (M = 1.725, SD = 0.803), and Master's degree (M = 2.047, SD = 0.969, *p* = 0.022) comparative to those who completed a 4-year college degree (M = 1.764, SD = 0.853) at the *p* < 0.05 level. Those with a Master's degree had higher mean scores, meaning a lower usefulness score than the other education levels.

### Download the exposure notification system app

For the question “Please indicate how likely you are to download (app) the Exposure Notification app if one of the below people or organizations asks you to,” there were five answer categories: healthcare provider; employer or school; city, country, or state public health department; federal public health authorities; and app store provider (such as Google or Apple). There were no statistically significant differences between the demographic groups for “city, county, or state public health department” or “Federal public health authorities.” For *healthcare providers*, there were significant differences between race/ethnicities [*F*_(3, 834)_ = 3.648, *p* = 0.012]. There were also group differences between the gender groups for *employer or schoo*l [*F*_(2, 835)_ = 5.232, *p* = 0.006] and group differences between age [*F*_(3, 832)_ = 2.692, *p* =0.045], education level [*F*_(4, 829)_ = 6.706, *p* ≤ 0.001], and ethnicity [*F*_(3, 834)_ = 3.756, *p* = 0.011] for likelihood to download app if asked by *app store providers*.

The Tukey's *post-hoc* analysis showed that for *healthcare providers*, the mean score for African Americans (M = 1.35, SD = 0.559, *p* = 0.010) was statistically significantly lower (i.e., more likely to download) than for the ethnic category “other” (M = 1.817, SD = 1.048). The Tukey's *post-hoc* test revealed that the mean scores of females (M = 1.55, SD = 0.903, *p* = 0.004) were statistically significantly different than males (M = 1.78, SD = 1.103), with the male group having higher mean scores than females for the question regarding *employer or school*.

Under “app store provider,” Tukey revealed mean differences to be significantly higher for white (M = 3.05, SD = 1.365, *p* = 0.005) compared to African Americans (M = 2.51, SD = 1.273) ethnicity, suggesting that African Americans in this sample were significantly more likely to download the app if asked by an app store provider.

Still under “app store provider,” the Tukey's *post-hoc* test for education showed that those with a terminal degree (PhD, MD, JD, etc.) (M = 3.42, SD = 1.269) had a significantly higher mean score, or lower likelihood of downloading the app, compared to those with some college (M = 2.77, SD = 1.343, *p* < 0.001), those who completed college (M = 2.93, SD = 1.423, *p* = 0.004), and those with a high school diploma or GED (M = 2.39, SD = 1.358, *p* < 0.001). Those with a high school degree (M = 2.39, SD = 1.358, *p* = 0.44) also had a significantly lower mean score than those with a Master's degree (M = 3.08, SD = 1.328). Although there was shown to be significant in mean scores for age under “app store provider,” the Tukey's *post-hoc* test did not reveal significance at the 0.05 level between age quartiles.

### Notify other service users

The five answer categories (healthcare provider; employer or school; city, country, or state public health department; federal public health authorities; and app store provider) applied as well to the question “If you were to test positive for COVID-19 today, how likely are you to let (app), and the Exposure Notification Service, notify other service users if one of the below people or organizations asks you to?” There were no statistically significant differences between the groups for “healthcare provider;” “city, country, or state public health department;” or “federal public health authorities.” For user likelihood to allow the app to notify other service users if asked by their employer or school, there were significant differences between gender [*F*_(2, 835)_ = 4.570, *p* = 0.011]. There were also group differences between age [*F*_(3, 832)_ = 6.591, *p* < 0.001] and education [*F*_(4, 829)_ = 4.260, *p* = 0.002], if asked by an app store provider (such as Google or Apple).

The Tukey's *post-hoc* analysis showed that the mean scores were statistically significantly lower for females (M = 1.38, SD = 0.855, *p* = 0.009) compared to males (M = 1.58, SD = 1.031) for the question asking about the likelihood of allowing the app to notify other service users if an employer or school asked. Under “app store provider,” the Tukey's *post-hoc* test revealed that those participants with a terminal degree (M = 3.21, SD = 1.496) were less likely to allow the app to notify other service users compared to those with some college (M = 2.60, SD = 1.371, *p* = 0.004) and those with a college degree (M = 2.71, SD = 1.523, *p* = 0.008), with a statistically significantly higher mean score. Also under “app store provider,” the age quartile 18–36 years (M = 2.50, SD = 1.462) with lower mean scores had a higher likelihood of allowing the app to notify service users than quartiles 50–61 years (M = 3.02, SD = 1.473, *p* = 0.002) and over 61 years (M = 3.04, SD = 1.501, *p* = 0.002).

### Send exposure notifications

When asked the question, “Please indicate how likely you are to let (GuideSafe™), a COVID-19 Exposure Notification Service, send your exposure notifications to the following individuals or organizations,” there were five categories as possible answers: healthcare provider; employer or school; city, county, or state public health department; federal public health authorities; and app store provider (such as Google or Apple). There was statistical significance of <0.05 for answer choices “your employer or school,” with group differences between age [*F*_(3, 832)_ = 4.738, *p* = 0.003] and ethnicity [*F*_(3, 834)_ = 3.510, *p* = 0.015] and “app store provider,” with group differences between age [*F*_(3, 832)_ = 4.991, *p* = 0.002] and education [*F*_(4, 829)_ = 5.209, *p* < 0.001].

The Tukey's *post-hoc* test revealed that the mean difference in age quartile over 61 years (M = 1.91, SD = 1.197) for the answer choice “your employer or school” was significantly higher than age quartiles 18–36 years (M = 1.61, SD = 0.940, *p* = 0.023) and 37–49 years (M = 1.53, SD = 1.018, *p* = 0.002). The answer choice “your employer or school” also had borderline significance between the ethnic groups such as white (M = 1.73, SD = 1.095, *p* = 0.051) and African Americans (M = 1.41, SD = 0.758), where the mean score of AA was mildly significantly lower than white.

The Tukey's *post-hoc* analysis indicated that for the answer selection “app store provider,” the age quartiles 37–49 years (M = 2.76, SD = 1.552) were significantly lower than the age quartiles 50–61 years (M = 3.18, SD = 1.476, *p* = 0.019) and over 61 years (M = 3.20, SD = 1.515, *p* = 0.018). The Tukey's *post-hoc* analysis also showed that the mean scores for education for the selection “app store provider” were statistically significantly higher for those with a terminal degree (M = 3.37, SD = 1.471) compared to those with some college experience (M = 2.78, SD = 1.475, *p* = 0.006) and those who completed college (M = 2.84, SD = 1.521, *p* = 0.005).

## Discussion

While mean scores across survey questions were low, indicating high levels of satisfaction and trust in sharing their COVID-19 infection information anonymously, we found significant differences between some groups for some of the survey questions indicating that some demographic groups may have differing opinions than others. For example, those with a higher education rated the ENS to be less useful, less easy to use, and less satisfied than others. In addition, African Americans found the ENS to be easier to use and more useful than those in the white demographic group.

In terms of sharing exposure notifications with others and the likelihood of sharing exposure information if asked by varying entities, consistent with the CT literature, was the reluctance to share by some groups (4, 5). For example, the analysis revealed that the age group over 61 years may have a lower likeliness to share with an employer or school than the age groups 18–36 and 37–49 years, which has also been found in other studies (31). We also found that younger ages reported a higher likelihood to share that information with an app store provider than older groups, though all the groups reported a lower likelihood of sharing COVID-19 infection alerts with app store providers than with other entities.

At the time of this publication, the authors could not find any literature specifically on the association between education level and the likelihood to share COVID-19 information. More generally though, the literature is mixed on the likelihood to share health-related information when education level is considered (32–34). We found that education levels seemed to make a difference, with those with a terminal degree reporting a lower likelihood of sharing COVID-19 information with app store providers compared to those with some college experience and those who completed college.

Whereas other studies suggest the importance of privacy as a factor in app usage (35), our results get at a deeper level as to specific contributors. We found that ethnicity and age may be critical factors for trust in sharing personal COVID-19 infection information, even if that information is sent to and received anonymously by public health institutions, employers, schools, healthcare providers, and technology designers that may want to consider these findings as they construct technologies and perform outreach campaigns aimed at reducing infection rates with the ENS and related technologies.

The findings from this study should be considered among its limitations. Because of the privacy-preserving nature of the (GuideSafe™) app, we have no way of knowing the users and if they resided in (Alabama). Instead, we surveyed those from a COVID symptom monitoring website as a means to capture those who have and have not used the (GuideSafe™) app and doing so may have led to selection bias. However, we have no way of distinguishing between survey participants that only downloaded the (GuideSafe™) app vs. those that downloaded and used the (GuideSafe™) app. Even though these findings are consistent with the literature, this may have led to selection bias and may limit the generalizability to broader populations.

## Conclusion

The (GuideSafe™) system was one integrated component of comprehensive education and work re-entry strategy across (Alabama) that reached a broad user base. Different users across the different demographic groups, including age, gender, education level, and ethnicity, perceive the sharing of information about their communicable disease exposures differently. Furthermore, these results show that these factors play a role in which types of organizations (i.e., healthcare, government, education, and tech companies) individuals are willing to share their communicable disease exposure information. Finally, user perceptions about the ease of use, satisfaction, and usefulness of the Exposure Notification Systems (ENSs) differ across the demographic groups, with education level playing a key role across usability measures. Public health institutions, employers, schools, healthcare providers, and technology designers may want to consider these findings as they construct technologies and perform outreach campaigns aimed at reducing infection rates with the ENS and related technologies.

## Clinical relevance statement

Lessons learned from swift technology developments during COVID-19 are translatable to other communicable diseases. Public health institutions and healthcare providers may benefit from using crowdsourced information about exposure notifications willingly shared by citizens. Global benefits include the ability to reduce exposure among the general population. More targeted benefits include reducing exposure among healthcare workers. General user sentiment is that these healthcare organizations and workers should be good information steward and apply strict procedures and control to preserve the privacy of individuals who report being at risk of exposure to communicable diseases.

## Data availability statement

The IRB, as approved, does not allow for data sharing. Please contact the corresponding author for further information.

## Ethics statement

The studies involving human participants were reviewed and approved by the University of Alabama at Birmingham institutional review board (IRB). Written informed consent was not required and the IRB reviewer determined this project is not subject to FDA regulations and is not Human Subjects Research.

## Author contributions

All authors listed have made a substantial, direct, and intellectual contribution to the work and approved it for publication.

## Funding

This study was funded through CARES ACT funding from the state of Alabama.

## Conflict of interest

The authors declare that the research was conducted in the absence of any commercial or financial relationships that could be construed as a potential conflict of interest.

## Publisher's note

All claims expressed in this article are solely those of the authors and do not necessarily represent those of their affiliated organizations, or those of the publisher, the editors and the reviewers. Any product that may be evaluated in this article, or claim that may be made by its manufacturer, is not guaranteed or endorsed by the publisher.

## Footnotes

^1^ https://www.census.gov/quickfacts/AL

^2^ https://data.census.gov/cedsci/table?q=S2801&g=0400000US01&tid=ACSST1Y2019.S2801
