# Corrigendum: User perceptions about sharing exposure notification information for communicable diseases

**DOI:** 10.3389/fdgth.2023.1157699

**Published:** 2023-07-11

**Authors:** Benjamin Schooley, Sue S. Feldman

**Affiliations:** ^1^ College of Engineering and Computing, University of South Carolina, Columbia, SC, United States; ^2^ Department of Health Services Administration, University of Alabama at Birmingham, Birmingham, AL, United States

**Keywords:** mobile platforms, privacy, pandemic response, exposure notification, contact tracing, communicable disease response

A Corrigendum on User perceptions about sharing exposure notification information for communicable diseases By Schooley B and Feldman SS. (2022) Front. Digit. Health 4:926683. doi: 10.3389/fdgth.2022.926683

**Incorrect Data Availability Statement**

In the published article, there was an error in the **Data Availability statement**. The authors did not adequately take into account restrictions put into place by the institutional review board (IRB) requirements on human subjects privacy. Not all data can be shared due to privacy restrictions. The original statement read: “The raw data supporting the conclusions of this article will be made available by the authors, without undue reservation.” The corrected Data Availability statement appears below:

“The IRB, as approved, does not allow for data sharing. Please contact the corresponding author for further information.”

**Incorrect Funding Statement**

In the published article, there was an error in the **Funding statement.** The name of the state “Alabama” was originally removed on purpose during the blind review process. However, the name was inadvertently left out in the final publication, an oversight that happened during the copyediting process. The original statement read: “This study was funded through CARES ACT funding from the state of (blinded for review).” The corrected Funding statement appears below:

“This study was funded through CARES ACT funding from the state of Alabama.”

**Incorrect Acknowledgements Statement**

In the published article, there was an error in the **Acknowledgements statement.** Acknowledgements to key individuals were inadvertently left out. We have added acknowledgments based on two individuals who worked on the app that was used for this study and should have been recognized for their work. Similarly, the Alabama Department of Public Health should have also been acknowledged but were inadvertently not mentioned. The original statement read: “Acknowledgment is given to Lincoln Laboratory, Massachusetts Institute of Technology for permission to adapt and use their survey instrument (communication dated 26 January 2021).” The corrected Acknowledgements statement appears below:

“The authors gratefully acknowledge the work of Brian Rivers and Rajesh Pillai for their leadership in development of the GuideSafe app. The authors also acknowledge the Alabama Department of Public Health for their support in development and dissemination of the GuideSafe app across the state of AL. Acknowledgment is also given to Lincoln Laboratory, Massachusetts Institute of Technology for permission to adapt and use their survey instrument (communication dated 26 January 2021).”

**Incorrect Ethics Statement**

In the published article, there was an error in the **Ethics statement.** This section previously stated: “The studies involving human participants were reviewed and approved by University of Alabama at Birmingham. Written informed consent for participation was not required for this study in accordance with the national legislation and the institutional requirements.” The corrected Ethics statement appears below:

“The studies involving human participants were reviewed and approved by the University of Alabama at Birmingham institutional review board (IRB). Written informed consent was not required and the IRB reviewer determined this project is not subject to FDA regulations and is not Human Subjects Research.”

The authors apologize for these errors and state that they do not change the scientific conclusions of the article in any way. The original article has been updated.

