# Supplementary material for: User perceptions about sharing exposure notification information for communicable diseases
Source: Front Digit Health. 2022 Jul 28;4:926683. doi: 10.3389/fdgth.2022.926683 (PMC9366094; doi:10.3389/fdgth.2022.926683)
Supplement: Supplementary file 1 [file Table_1.DOCX]

Supplemental Material: Survey Questions

| **Awareness (yes/no/not sure)** |
| --- |
| Have you heard of contact tracing? |
| ^+^ Please choose the answer that best describes your understanding contact tracing   - Contact tracing is the process of be followed by someone using my phone as a mechanism - Contact tracing is a confidential process of identifying people who may have come into contact with an infected person and subsequent collection of further information about these contacts, such as with COVID-19 - Contact Tracing is the government tracking my movements - Contact Tracers are people who come to my home and isolate me from my family when they find out that I have come in contact with someone with a contagious disease, such as COVID-19 |
| ^+^Where have you heard of contact tracing? (government communication, traditional media, social media, friends/family, app store, smartphone notification, other [please specify]) |
| Have you heard of COVID-19 exposure notifications |
| ^+^Where have you heard of COVID-19 exposure notifications? (government communication, traditional media, social media, friends/family, app store, smartphone notification, other [please specify]) |
| **General experience with COVID-19 information (5 point Likert Scale 1-definitely yes to 5-prefer not to say)** |
| I have regular visits/check-ups with medical professionals (at least once a year) |
| I regularly seek out medical information on the Internet (websites, social media, app; a few times a month or more) |
| I know someone that has/had a severe case of COVID-19 |
| I know someone that has/had a mild case of COVID-19 |
| **Health Attitudes and behaviors (5 point Likert Scale 1-strongly agree to 5-NA/prefer not to say)** |
| I generally understand when I am in a high-risk situation for contracting COVID-19 |
| I know the COVID-19 risk level my state has assigned to my town/city |
| The risks from COVID-19 have been overstated in the media |
| My household is not in danger of contracting COVID-19 |
| I practice all CDC/ medical professional recommendations for COVID-19 |
| **COVID-19 circumstances (5 point Likert Scale 1-definitely yes to 5-NA/prefer not to say)** |
| I believe I am high-risk for COVID-19 complications |
| I believe a loved one is high-risk for COVID-19 complications |
| I have experience living with or caring for someone with COVID-19 |
| I know someone in my building or apartment complex was positive for COVID-19 |
| **Opinions about participating in contact tracing or exposure notification (5 point Likert Scale 1-extremely likely to 5-extremely unlikely)** |
| If you were to test positive for COVID-19, you may be asked to provide the names and phone numbers or addresses of the people you have been physically close to in the last two weeks so those people can be informed of their risk. Please indicate how likely you are to share that information if one of the below people or organizations calls you on the phone and asks you to |
| - Your doctor or healthcare provider |
| - Any doctor or nurse |
| - Your employer or school |
| - City, county, or state public health department |
| - Federal public health authorities (such as CDC) |
| - App store provider (such as Google or Apple) |
| **Exposure notification availability and use (yes/no/not sure)** |
| Some states are deploying exposure notifications that can tell you on your smartphone if you've come into contact with a potentially infectious person. Are you aware of COVID-19 exposure notification app called [blinded for review] in ]state blinded for review]? |
| ^+^ Have you enabled or downloaded your state's COVID-19 exposure notifications? |
| These COVID-19 exposure notifications are built to keep users anonymous. Are you comfortable with the level of anonymity they provide? |
| **mHealth App Usability Questionnaire (MAUQ) Only displayed if answered yes to “enabled or downloaded” question (5 point Likert Scale 1-very much to 5-none)** |
| **Ease of Use** |
| The app was easy to use |
| It was easy for me to learn to use the app. |
| The navigation was consistent when moving between screens. |
| The interface of the app allowed me to use all the functions (such as connecting to Healthcheck, reporting a positive test, reading information) offered by the app. |
| Whenever I made a mistake using the app, I could recover easily and quickly. |
| **Interface and Satisfaction** |
| I like the interface of the app. |
| The information in the app was well organized, so I could easily find the information I needed. |
| The app adequately acknowledged and provided information to let me know the progress of my action |
| I feel comfortable using this app in social settings. |
| The amount of time involved in using this app is appropriate. |
| I would use this app again. |
| Overall, I am satisfied with this app. |
| **Usefulness** |
| The app is useful for my health and well-being. |
| The app improved my access to COVID-19 guidance.* |
| The app helped me manage my COVID-19 risk.* |
| This app has all the functions and capabilities I expected it to have. |
| I could use the app even when the Internet connection was poor or not available. |
| This app provided an acceptable way to address COVID-19 risks, such as accessing information, performing self-assessment, and reporting risks to others.* |
| **App download likelihood (5 point Likert Scale 1-extremely likely to 5-extremely unlikely)** |
| COVID-19 exposure notifications can alert you if you have previously been in close proximity to someone who later tests positive for COVID-19. Imagine you are asked to **download**  GuideSafe, a COVID-19 exposure notification app. Please indicate how likely you are to do so if one of the below people or organizations asks you to. (Please answer even if you have already downloaded or enabled COVID-19 exposure notifications.)   - Your doctor or healthcare provider - Any doctor or nurse - Your employer or school - City, county, or state public health department - Federal public health authorities (such as the CDC) - App store provider (such as Google or Apple) |
| Please indicate how likely you are to download GuideSafe, a COVID-19 exposure notification app on your own, without anyone asking you directly |
| Please indicate how likely you are to enable COVID-19 exposure notifications on your smartphone |
| **COVID-19 test result upload likelihood (5 point Likert Scale 1-extremely likely to 5-extremely unlikely)** |
| COVID-19 exposure notifications can alert other users of those exposure notification services that they have been physically close to someone who later tests positive for COVID-19, without sharing identifying information, but only if the potentially infectious person lets the service notify them.  If you were to test positive for COVID-19 today, how likely are you to **let [blinded], an exposure notification service, notify other service users** if one of the below people or organizations asks you to?   - Your doctor or healthcare provider - Any doctor or nurse - Your employer or school - City, county, or state public health department - Federal public health authorities (such as the CDC) - App store provider (such as Google or Apple) |
| Please indicate how likely you are to let [blinded], a COVID-19 exposure notification service, notify other service users on your own, without anyone asking you directly.   - Your doctor or healthcare provider - Any doctor or nurse - Your employer or school - City, county, or state public health department - Federal public health authorities (such as the CDC) - App store provider (such as Google or Apple) |
| Please indicate how likely you are to let ]blinded], a COVID-19 exposure notification service notify other service users if **data or information about those notifications or contacts is not viewable by anyone**. |
